# Protocol for a multicentre randomised controlled trial of the Pharmacy Homeless Outreach Engagement Non-medical and Independent Prescriber (PHOENIx) intervention for people facing severe and multiple disadvantages

**DOI:** 10.1136/bmjopen-2025-106640

**Published:** 2025-11-23

**Authors:** Richard Lowrie, Andrew McPherson, Jane Moir, Emma McGilvery, Katherine Vickery, Jen O’Loan, Gordon Rushworth, Vibhu Paudyal, Alex Adam, Elaine Thomson, Alison Rowe, Hannah Ali Akbar, John Murphy, John Budd, Fiona Raeburn, Trudi Marshall, Kirsty Nelson, Zofia Garstka, Emma McKinney, Lauren Melville, Graeme Duncan, Zoe Breingan, Sarah Johnsen, Andrew Stoddart, Steff Lewis, Andrea E Williamson, Jack Lilley, Tracy Orr, Michael Orr, Shona Kelly, Mairi Macaulay, Alison Maclean, Heather Kennedy, Andrea Sutherland, Gillian MacLean, Magda Rosinska, Carlos Dos Santos, Kelly Esson, Craig Robertson, Jill Carnegie, Mariangela Alejandro Cortez, Marion Orr

**Affiliations:** 1 Centre for Homelessness and Inclusion Health, The University of Edinburgh, Edinburgh, UK; 2 Pharmacy Services, NHS Greater Glasgow and Clyde, Glasgow, UK; 3 NHS Ayrshire and Arran, Ayr, UK; 4 Department of Medicine, Hennepin County Medical Centre, Minneapolis, Minnesota, USA; 5 NHS Highland, Inverness, UK; 6 Robert Gordon University School of Pharmacy and Life Sciences, Aberdeen, UK; 7 Highland and Islands Pharmacy Education and Research, NHS Highland, Inverness, UK; 8 King’s College London, London, UK; 9 NHS Lothian, Edinburgh, UK; 10 NHS Grampian, Aberdeen, UK; 11 NHS Lanarkshire, Lanarkshire, UK; 12 The Steeple Church, Dundee, UK; 13 Simon Community Scotland, Edinburgh, UK; 14 Edinburgh Health Services Research Unit, The University Of Edinburgh, Edinburgh, UK; 15 Edinburgh Clinical Trials Unit, University of Edinburgh, Edinburgh, UK; 16 GPPC, School of Medicine, Dentistry and Nursing, MVLS, University of Glasgow, Glasgow, UK; 17 Simon Community Scotland, Perth, UK; 18 Simon Community Scotland, Scotland, UK; 19 New Start Highland, Inverness, UK; 20 Alcohol and Drugs Action, Aberdeen, UK; 21 Turning Point Scotland, South Lanarkshire, UK

**Keywords:** PUBLIC HEALTH, Social Support, Primary Health Care, Randomized Controlled Trial, Health Equity

## Abstract

**Introduction:**

People experiencing severe and multiple disadvantage (SMD: homelessness, substance use and criminal offending) have multiple intersecting unmet health and social care needs and high mortality rates, often due to street-drug overdose. Pilot randomised controlled trials (RCTs) suggest an integrated, holistic, collaborative outreach intervention (Pharmacy Homeless Outreach Engagement Non-medical Independent Prescribing Rx (PHOENIx)) involving generalist-trained pharmacists, nurses or General Practitioners accompanied by staff from third sector homeless organisations may improve outcomes, including reducing overdose.

**Methods:**

Multicentre, parallel group, prospective RCT with parallel economic and process evaluation. Set in six areas of Scotland, UK, 378 adults with SMD will be recruited and randomised (stratified by setting and previous non-fatal overdoses) to PHOENIx intervention in addition to usual care (UC) or UC. Aiming to meet participants weekly for 9–15 months, PHOENIx teams assess and address health and social care needs while referring onwards as necessary, co-ordinating care with wider health and third sector teams. During a person-centred consultation, in the participants’ choice of venue, and taking account of the participant’s priorities, the NHS clinician may prescribe, de-prescribe and treat, for example, wound care, and refer to other health services as necessary. The third sector worker may help with welfare benefit applications, social prescribing or advocacy, for example, securing stable housing. Pairings of clinicians and third sector workers support the same participants. The primary outcome is time to first fatal/non-fatal street-drug overdose at nine months. Secondary endpoints include health-related quality of life, healthcare use and criminal justice encounters. A health economic evaluation will assess cost per quality adjusted life year of PHOENIx relative to standard care. A parallel qualitative process evaluation will explore the perceptions and experiences of PHOENIx, by participants, stakeholders and PHOENIx staff.

**Analysis:**

The primary and other time-to-event secondary outcomes will be analysed by Cox proportional hazards regression.

**Ethics and dissemination:**

IRAS number 345246, approved 23/10/2024 by North of Scotland Research Ethics Service. Results will be shared with participants, third sector homelessness organisations, health and social care partnerships, then peer-reviewed journals and conferences worldwide, from the first quarter of 2027.

**Trial registration number:**

ISRCTN12234059 registered on 20/2/2025 (ISRCTN).

**STRENGTHS AND LIMITATIONS OF THIS STUDY**

- Pre-registered, definitive, multicentre individually randomised controlled trial (RCT) with parallel process and economic evaluation, informed by feasibility studies and pilot RCTs.
- Largest UK community-based intervention trial involving people experiencing homelessness, substance use and a history of criminal offending (Severe and Multiple Disadvantage).
- First definitive RCT testing partnership between NHS Clinicians (Pharmacists, Nurses, General Practitioners) and third sector homelessness organisations, addressing wider determinants of health.
- Parallel economic and qualitative process evaluations will inform affordability and implementability.
- The context and findings may not be generalisable outwith the UK.

## Introduction

 Worldwide, people experiencing severe and multiple disadvantage (SMD) (homelessness, criminal justice involvement and substance use (SMD)) struggle to access healthcare and have worse outcomes than people living in mainstream society.^1^,^3^ A majority are frail,^4^ have suffered adverse childhood experiences^5^ and are re-traumatised by their current living conditions without protective social relationships.^6 7^

Multiple barriers limit access to primary healthcare for people who have SMD, for example, stigma, discrimination, fragmented care pathways and healthcare professional skill-mix.^8^,^10^ People who have SMD are on average 43 years old, with numbers of long-term conditions on a par with 85-year-olds who live in their own homes. However, unlike multimorbid 85-year-olds, half of their conditions are untreated.^46 10^,^12^ The work involved in self-management has a profound impact on well-being and daily activities for people who have SMD.^13^ When asked to rate their quality of life, one third describe health states equivalent to scores the general population would regard as ‘worse than death’.^4 12^

Substance use has been shown to be both a cause and consequence of homelessness.^14^ In Scotland, two-fifths of deaths in people experiencing homelessness are attributed to street poly-drug use and drug death rates in the general population (22.4 per 100 000) are second only to North America.^15^,^17^ A well-established evidence base confirms Opiate Substitution Treatment (OST) protects against drug-related mortality but is insufficient to reduce escalating drug death rates.^18 19^ People who have SMD tend to continue to overdose repeatedly (often with a combination of prescribed OST and street drugs) despite receiving recommended doses of OST,^4^ suggesting a need for additional, complementary, upstream interventions to reduce overdose risk.

People who have SMD are under-represented in previous studies including those generating evidence for the effectiveness of OST or heroin-assisted treatment.^18^,^20^ To date, interventions targeting people experiencing homelessness with or without substance dependency and criminal offending behaviours have not addressed untreated multimorbidities and social determinants of health in tandem.^21^,^23^ Existing evidence suggests that people who have SMD prefer integrated services, rather than attending multiple, fragmented services and repeatedly re-telling often traumatic histories.^7 22 23^ People experiencing SMD have what some describe as ‘no hope for any kind of a decent life’.^7^

Theories of behaviour change and interventions for people with substance dependency suggest the importance of relationships in fostering recovery.^24 25^ Staff in third sector (charity) homelessness organisations are often the first point of contact and support for people facing SMD, providing close relationships and trauma-informed practical and emotional support.^26 27^

Given the extent and range of health and social care challenges faced by people who have SMD,^34 6 7 10^,^13^ research is needed to understand whether holistic, trauma-informed interventions delivered by combined third sector and health teams improves outcomes, including reducing drug deaths.

Following extensive pilot and feasibility studies, a collaborative NHS clinician and third sector homelessness worker intervention (Pharmacy Homeless Outreach Engagement Non-medical Independent Prescribing Rx (PHOENIx)) showed promise as a means of reducing the risk of overdose and improving quality of life for people facing SMD.^47 12 28^,^30^ PHOENIx offers a relationship-based supportive bridge to connect people with services, taking account of the person’s priorities and adopting a trauma-informed approach.^1931^,^33^ The generalist clinician (pharmacist, nurse or General Practitioner (GP)) and third sector homelessness worker outreach together in pairs, once weekly on average, meeting people who have SMD, outwith health service buildings. PHOENIx offers assessment, treatment and referral for health problems (including substance dependence) alongside emotional and practical support for wide-ranging problems. PHOENIx offers co-ordinated, integrated trauma-informed care in addition to care as usual.

We hypothesise that PHOENIx reduces the risk of overdose and improves other outcomes of importance to people facing SMD, and is cost effective and implementable.

### Research questions

#### Primary research question

Does PHOENIx reduce occurrence of fatal or non-fatal drug overdose (confirmed by self-report, a witness or documented in health/social care/third sector case records) from randomisation until 9 months (or as close as possible to 9 months), assessed as a time-to-event outcome for people who have SMD?

#### Secondary research questions

In comparison with people with SMD in the usual care (UC) arm of the study, at follow-up (9 months or as close as possible to 9 months) does PHOENIx:

- Improve health-related quality of life (EQ-5D-5L)?
- Delay time to event and reduce the number of emergency department presentations?
- Delay time to event and number of ambulance call outs?
- Delay the time to event and reduce the number of hospitalisations?
- Increase treatment uptake for physical and mental health problems and problematic drug use?
- Decrease street-drug use?
- Decrease the number of overdoses?
- Increase primary care contacts attended and decrease primary care missed appointments.
- Increase out-patient clinic attendance and decrease out-patient clinic missed appointments.
- Increase GP, Alcohol and Drug Recovery Service (ADRS) and mental health team registration.
- Increase ADRS and mental health team attendance.
- Increase the uptake of residential drug rehabilitation?
- Reduce all-cause and cause-specific mortality?
- Improve scores on validated measures of health:
  - Body Mass Index in normal range?
  - Blood pressure in normal range?
  - Forced Expiratory Volume in 1 s, Forced Expiratory Volume in 6 s and lung age?
  - Modified MRC breathlessness scale?
  - PHQ-4 depression and anxiety screening?

Does PHOENIx lead to an increase in regular meals?

Does PHOENIx lead to an increase in regular exercise?

Does PHOENIx

- Reduce involvement with the criminal justice system (criminal behaviour, criminal offending, police involvement (cautions, arrests), imprisonment, remand)?
- Delay time to event or reduce the number of criminal justice system contacts?

Does PHOENIx increase community integration and functioning?

- Increase the uptake of vocational (paid or voluntary) employment or training?
- Increase the uptake of social prescribing opportunities.

Does PHOENIx effect housing utilisation and support?

- Increase the uptake of emergency/temporary accommodation (if street homeless)?
- Increase tenancy sustainment?
- Decrease the number of moves between accommodation settings.
- Decrease intensity or level of support received?

Does PHOENIx impact on social services use?

- Change the uptake of welfare benefits?
- Increase the total income from benefits?

Does PHOENIx provide a cost-effective intervention relative to UC as observed over the 9-month trial period, in assisting people with SMD:

- From a health and social care perspective (under NICE reference case criteria)^34^?
- From a social return on capital perspective (additionally accounting for third sector, criminal justice and welfare payments)?

How is the PHOENIx intervention perceived and experienced by people with SMD, external stakeholders and staff?

- What (implementation and contextual) factors facilitate or inhibit delivery and achievement of intended outcomes?

## Methods

### Patient and public involvement

People who have SMD were involved in the early qualitative evaluation of PHOENIx, confirming the acceptability and the potential that it improved health.^28^ Subsequently, people experiencing homelessness with or without substance dependency were interviewed about the prospect of a RCT^35^ and others were asked about their priorities for research.^36^ In both studies, the prospect of a study to evaluate the health effects of combined health and social care outreach interventions was supported. People who had SMD have been consulted throughout the pilot studies and informed the planned PHOENIx intervention.^31^,^33^

### Study design and setting

The study is an open, multicentre, parallel group, prospective RCT with parallel economic analysis and process evaluation. Scotland has 14 territorial health board areas, collectively responsible for healthcare and health improvement for the population (5.5 million) spread across urban, remote and rural, and coastal areas. The study will be set in six health board areas: Lothian; Tayside; Grampian; Highland; Lanarkshire; and Ayrshire and Arran. Within each of these areas, researchers will be recruited from local third sector homelessness organisations. Also in each area, PHOENIx teams (NHS generalist clinicians (pharmacists, nurses or GPs) and third sector homelessness workers) will be recruited from NHS boards and third sector homelessness organisations, respectively.

### Participant eligibility

Table 1 describes participant inclusion criteria. At the time of assessment for eligibility, participants will be: homeless^37^ with at least one previous street-drug overdose (self-report or witnessed by another person); and with a criminal justice history.

**Table 1:** Inclusion and exclusion criteria

Inclusion	Exclusion
Adults (aged 18 years and over) experiencing homelessness as categorised by the ETHOS typology^37^ ≥1 non-fatal drug overdose (confirmed by self-report, a witness or documented in health/social care/HVCSE case records) ≥1 self-reported previous criminal justice encounter (arrested /cautioned /imprisoned)	Living in residential accommodation with 24-hour medical care Posing a known safety risk to self/others Lacking capacity to consent (although if due to intoxication, researchers may offer recruitment at a later date)

### Participant identification, informed consent and recruitment

**Figure 1:**
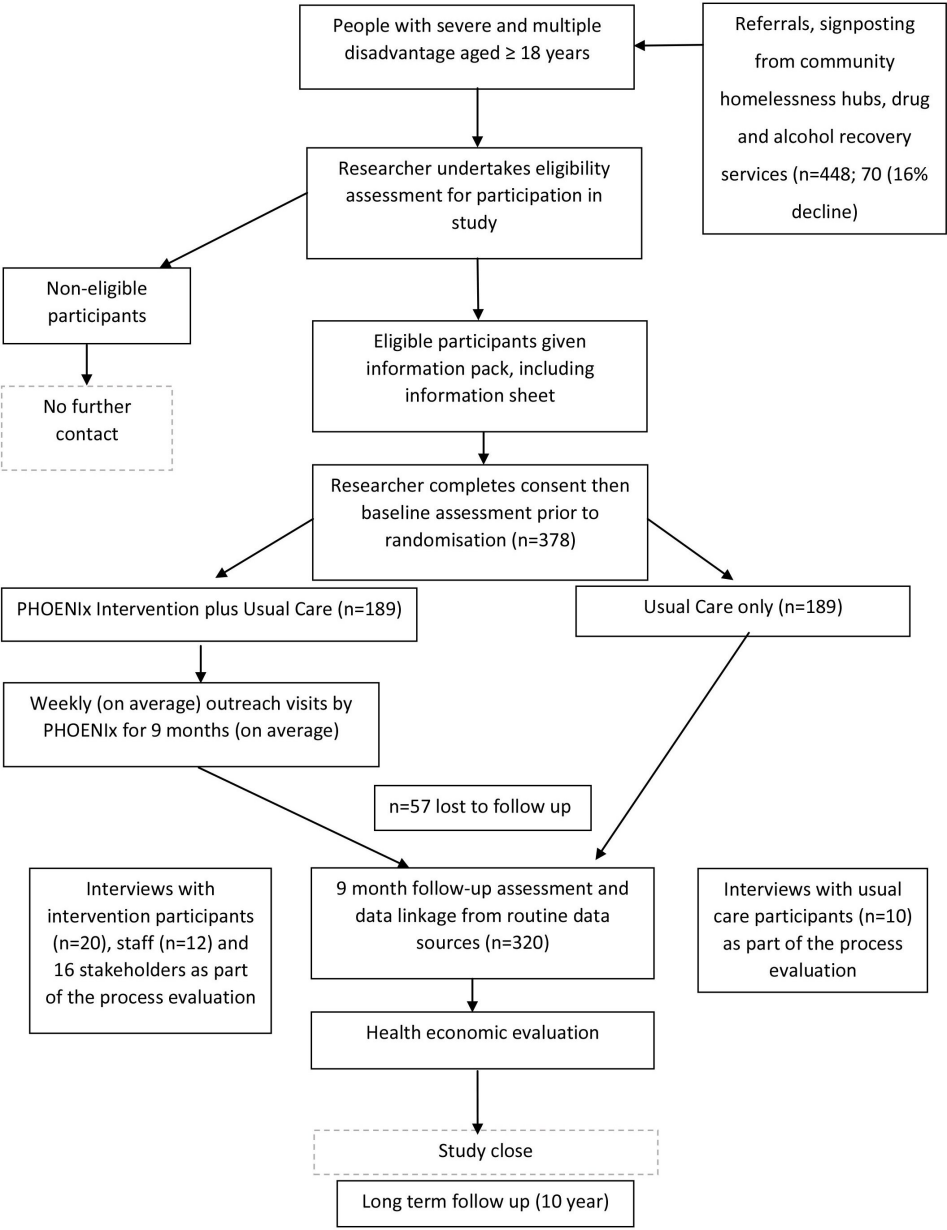
Participant recruitment.

Posters describing the study and participant eligibility will be displayed in homelessness hubs and emergency/temporary accommodation venues. Researchers will meet with third sector, health and social care staff to inform them of the study and provide them with an opportunity to ask questions about it. Potential participants will then be identified and approached by health and third sector personnel, including accommodation providers (all of whom routinely care for people facing SMD) who will hand potential participants study information in advance of researchers explaining the study in more detail. Figure 1 summarises participant recruitment, with the target (n=378) expected by July 2025 (online supplemental file 4).

At least 24 hours later, after potential participants have had a chance to read the English language paper version of the participant information sheet (PIS) or have this explained to them, researchers (third sector staff) will approach potential participants in pairs, for safety reasons, explaining the study and, if appropriate, obtaining signed informed consent. Online supplemental material file 1 shows the participant consent form. Consent includes permissions to access all relevant health, social care and criminal justice records.

For potential participants whose first language is not English, an interpreting service will be asked to read and explain the PIS. All researchers will have completed Good Clinical Practice (GCP) training and all will have undergone specific training in informed consent and capacity. Research managers (AM and JM) have experience of consenting participants into RCTs through previous and similar studies. Researchers will observe the Adults with Incapacity (Scotland) Act 2000 for the identification and exclusion of those who may lack capacity to consent to the study.

At the start of the informed consent process, researchers will converse with potential participants and ask them the date and time to gauge orientation. Having a meaningful conversation and being orientated will help researchers gauge the capacity of the potential participant. Informed consent will be taken by researchers prior to commencing baseline data collection.

Participants will be offered a £20 shopping voucher at baseline, and at 9 months, and for qualitative interviews (see below), as a thank you for participating in the study.

### Sample size and statistical power calculations

Collecting 130 primary outcome events (fatal or non-fatal street-drug overdoses), the trial has 90% power (two-sided, log-rank test with a 5% CI) to detect a difference of 18% (assuming 53% in the U arm and 34% in the PHOENIx plus UC arm have a primary outcome event) in the proportion of participants experiencing overdose at 9 months. We estimate that we would be able to collect this number of outcomes by following 160 participants per group (320 in total) to 9 months. Allowing a 15% loss to follow-up, we will recruit 378 participants. This 15% loss to follow-up figure was derived from the PHOENIx pilot study, where 17 participants out of 116 were lost to follow-up (15%), who were still alive and had capacity at 9 months follow-up.^9^

### Baseline data collection

Immediately after obtaining participant consent, health and social care data will be collected during in-person assessments by researchers at baseline, aiming to collect a wide range of information (online supplemental material file 2). Before conducting assessments, researchers will receive training from research managers (AM and JM) who have experience leading recruitment from pilot studies. Baseline assessments will take place in person in participants’ temporary accommodation, third sector homelessness ‘hubs’, healthcare venues, the street or other low threshold service settings. Baseline interviews will take up to one hour to complete. Secondary data will be collected after interviews by researchers from participants’ health and social care records. These records are kept by General Practices, ADRS, hospitals and third sector organisations. They will be accessed by the study researchers who have permissions to collect these data.

### Randomisation

Immediately following baseline assessment, and in the presence of the participant, researchers will telephone a dedicated phone line at the Robertson Centre for Biostatistics University of Glasgow interactive voice response service (IVRS) to independently randomise. The IVRS will ask for the researcher’s identification, the participant’s code number and number of previous street-drug overdoses. Randomisation will be 1:1 to either PHOENIx plus UC, or UC only, and will be stratified by site and participant self-report of lifetime history of non-fatal street-drug overdoses, grouped as; 1 to 5, 6 to 10, 11 to 20 and more than 20, based on pilot studies.^9 17^

### Follow-up data collection

In person and secondary data for the primary and secondary outcomes will be collected approximately 9 months after baseline assessments (online supplemental material file 3). Criminal justice encounters will be obtained directly through data linkage using Police Scotland administrative records.

### Blinding

Due to the nature of the intervention, it is not possible to blind participants or PHOENIx intervention staff. Researchers collecting follow-up data in some of the settings will be blinded to participant allocation, and in other settings (where there are fewer participants and researchers), researchers will know participant allocation, because of having collected baseline data and randomised. The researchers will remain independent and not get involved with the direct care of participants except in any circumstances when there is an urgent need to do so. Circumstances when this might occur would be serious threatening mental health issues (plans of suicide, self-harm or harm to others), or in the event of a participant overdosing during an assessment. All such events will be recorded and brought to the attention of the Trial Steering Committee (TSC).

### Intervention

The PHOENIx intervention is described below using the TIDieR framework and checklist.^38^

### Background

Adapting to the participant’s pace, PHOENIx teams take account of the participant’s priorities after forming a list of health and social care problems identified during the first consultation. The participant will be asked to prioritise and during consultations, the NHS clinician assesses and addresses participants’ health problems by prescribing and referral to optimise treatments for a range of conditions when needed. During the same consultation, the third sector worker will assess and address the participant’s prioritised social care needs. The PHOENIx team will also provide harm reduction advice and supplies, for example, clean needles and injecting equipment, naloxone. The same pair of staff will aim to deliver the intervention weekly in person, with phone calls in addition (or instead), to a dedicated, low caseload of participants, for example, five per day.

### Why

PHOENIx is a complex intervention^39^ aiming to improve access and continuity of health and social care through trusting relationships. It aims to increase the uptake of treatments for multiple conditions and attendance at services while improving self-care and preventing deterioration in health through timely, immediate health and social care intervention on outreach. The rationale for joint health and third sector working is the diverse, unmet, multiple health and social care needs of the target group which cannot be addressed by either alone. Outreach is more likely to reach people facing SMD than static services.^26 40^

### Where

The PHOENIx team will assertively outreach to deliver the intervention in congregate accommodation housing multiple people experiencing homelessness, for example, hostels, B&Bs, low threshold third sector homeless drop-in hubs, the street or other low threshold communal venues, for example, soup kitchens.

### How

Before, during or after consultations, the NHS clinician will access the participant’s NHS clinical records on a laptop. Together with the information obtained during the consultation, the NHS clinician will have full awareness of current and previous diagnoses, treatments, referrals, scheduled, attended and missed appointments. Observations and actions will be recorded on paper forms before being transcribed onto the patient’s primary and/or acute care clinical records. In the same way, the third sector worker will access and update the participant’s third sector record (if there are previous contacts with the organisation delivering the intervention) after the consultation. Serial consultations will enable multiple health and social care problems to be prioritised by the participant, assessed and progress made, at the participant’s pace. Through multiple conversations and interventions, the PHOENIx team builds trusting therapeutic relationships, in the context of a trauma-informed approach to care.

### What

Working within the clinical governance framework provided by the person’s existing clinical team (if they have a registered GP and/or are known to other services eg, ADRS, or blood borne virus team), the clinician conducts a structured clinical assessment covering common physical and mental health problems, similar to the Baseline health assessment (online supplemental material file 2). The clinician will use their clinical and situational judgement, and complete validated screening tools as a means of assessing the participant’s health needs and status, for example, anxiety/depression (PHQ-4); modified Medical Research Council breathlessness score; cardiovascular disease (ASSIGN2); malnutrition universal screening tool. The clinician will assess and treat accordingly, which may include prescribing (for any health condition), de-prescribing and referral to a range of different health services as needed. The third sector worker will manage the participant’s wider needs, for example, benefits claim support, practical help, for example, household goods, advocacy, while liaising with the participant’s support worker. Participants will be offered help to attend appointments as required. The combination of health and third sector support is available at each consultation, but the participant’s priorities may focus on either, both or none, depending on their priorities and state of sobriety at the time.

### When and how much

PHOENIx teams aim to visit participants weekly, with consultations expected to last an hour on average. PHOENIx clinicians and third sector workers aim to visit five participants per day on average, depending on the distance travelled and availability of participants. The team aims to maintain contact with participants in their caseload, for up to 9 months. PHOENIx teams will use their judgement to gauge how much support each participant is offered, within the confines of their working day and caseload. Phone calls will supplement visits for participants who, in the opinion of the team and participant, need additional support between weekly visits, or if the participant cannot be located.

### Who provides the support?

As described previously,^4^ the PHOENIx clinician is an NHS registered health professional who is a generalist, with advanced clinical assessment skills and in most cases, also holds a non-medical independent prescribing qualification. This qualification enables the clinician to initiate, discontinue or modify the dose of any medicine within their competency. The clinician will be recruited from the local NHS primary care organisation. The PHOENIx third sector worker will be recruited from a local third sector homelessness charity and has experience of working with people who have SMD, to support their recovery through emotional and practical support. The clinician and third sector worker will always work together, in pairs. All staff (and researchers) are recruited on the basis of their commitment to providing emotional, clinical and practical support as part of a team. Staff will have street sense, respect, empathy and a non-judgemental attitude. Previous work has shown the importance of these attributes,^25^ trauma-informed approaches^27^ and integrated health and social support on outreach.^28 32 33^ The PHOENIx team’s relationship-based work is thought to facilitate participant motivation, fulfilling practical and emotional needs.^32^

Intervention staff expected to deliver PHOENIx are detailed in table 2. Staff will work in pairs and aim to retain contact with the same caseload of participants throughout the study.

**Table 2:** Staff and participant numbers

NHS boards	Clinical (WTE)	Third sector staff (WTE)	Participants (approx.)
Grampian	1	1	75
Ayrshire and Arran	1	1	75
Tayside	0.4	0.4	25
Lothian	2.5	2.5	128
Highland	0.5	0.5	35
Lanarkshire	0.5	0.5	40
Totals	5.9	5.9	378

WTE, Whole Time Equivalent.

### Intervention fidelity

This will be assessed by the research team, visiting PHOENIx teams on outreach, shadowing consultations to confirm the intervention is being delivered as planned, and noting any patterns suggesting the intervention is not being offered as planned.

### Usual care

All participants will continue to receive UC in their locality (health, third sector, social and any other care) throughout the study. The characteristic of UC in each setting will be collected by researchers.

## Outcomes

### Primary outcome

The primary outcome of the study is the first occurrence of fatal or non-fatal street-drug overdose from randomisation until 9 months assessed as a time-to-event outcome for participants receiving the PHOENIx intervention versus UC. This will be assessed by self-report, supplemented by information collected from participants’ clinical and third sector notes.

### Secondary outcomes

Secondary outcomes are given in table 3.

**Table 3:** Secondary outcome measures (from baseline to 9-month follow-up)

Clinical outcome measures	Drug and alcohol measures	Social measures
Improve health-related quality of life using the generic measure EQ-5D-5L Delay the time-to-event and reduction in the number of ED presentations Delay the time-to-event and reduction in the number of ambulance call-outs Delay the time-to-event and reduction in the number of hospitalisations Increase treatment uptake for physical and mental health problems Increase treatment uptake for problematic street-drug use Increase in primary care contacts attended and decrease in primary care missed appointments Increase in outpatient clinical attendance and decrease in outpatient clinic missed appointments Increase GP, ADRS and mental health team registration Increase in ADRS and mental health team attendance Reduce all-cause and cause-specific mortality Improvement in body mass index Improve blood pressure in the normal range Forced expiratory volume in 1 s, forced expiratory volume in 6 s and lung age Modified MRC breathlessness scale PHQ depression and anxiety scores	Decrease in street-drug use Decrease the number of overdoses Increase the uptake of residential drug rehabilitation	Increase in regular meals Increase in regular exercise Reduction in involvement in the criminal justice system (criminal behaviour, criminal offending, police involvement—cautions, arrests, imprisonment, remand) Increase time-to-event or reduce the number of criminal justice events Increase the uptake of vocational (paid or unpaid) employment or training Increase the uptake of social prescribing Increase the uptake of emergency/temporary accommodation (if street homeless) Increase in tenancy sustainability Decrease the number of moves between accommodation settings Decrease in intensity or level of support received Change in uptake of welfare benefits Increase the total income from welfare benefits

ADRS, Alcohol and Drug Recovery Service; ED, emergency department; GP, General Practitioner.

### Health economic evaluation

Economic analysis will be performed by health economists from Edinburgh Clinical Trials Unit (ECTU), with full details pre-specified in a health economic analysis plan prior to unblinding. This will include: a NICE reference case^34^ based 9 month within-trial cost utility analysis of PHOENIx relative to UC, reporting cost-per Quality Adjusted Life year (QALY) from an NHS and personal social services (PSSs) perspective. The probability of cost-efficiency will be reported at standard NICE thresholds (currently £20 k and £30 k per QALY).^34^ A further breakdown of the cost profile of people experiencing SMD using PHOENIx versus UC will be conducted in terms of NHS and PSS utilisation; criminal justice activities and welfare payments. PHOENIx programme costs will be estimated by a detailed activity-based costing exercise, with wider data extracted from routine clinical, Police Scotland and third sector records.

### Process evaluation

This evaluation will help to determine the potential barriers and facilitators to implementation of the PHOENIx intervention. The process evaluation will take the form of qualitative semi-structured interviews featuring stakeholders (a subset of participants n=30; PHOENIx staff in frontline and managerial roles n=12; and external stakeholders representing relevant housing, health, social care and other eg, domestic abuse support n=16). Interviews will be carried out by researchers from the University of Edinburgh, who are experienced in qualitative interviewing of people who have SMD. Participants will be offered a £20 shopping voucher as a thank you for their participation in the interviews. Interviews will be carried out in a therapeutic space (usually a counselling room in a third sector homelessness charity drop-in hub) for privacy and transcribed verbatim. Interview questions will be guided by the three main outcomes of the process evaluation: implementation (ie, the structures, resources and processes through which delivery is achieved), mechanisms of impact (especially participants’ and other stakeholders’ perceptions of and interactions with the intervention) and context (particularly factors facilitating or inhibiting delivery and attainment of intended outcomes).

### Analysis

Statistical analyses will be undertaken by staff from ECTU using a detailed statistical analysis plan. The primary outcome, and other time-to-event secondary outcomes, will be analysed by Cox proportional hazards regression, and results will be presented as HRs with 95% CIs. If the collected data violate the proportional hazards assumption, then we will use restricted mean survival analysis. Kaplan-Meier plots will also be produced, to allow exploration of how the intervention is taking effect over time. We aim to limit missing data as much as possible from the outset of the study. However, the plan to deal with missing data is outlined in the statistical analysis plan with planned covariate adjustment.

Qualitative data from the process evaluation will be analysed by researchers from the University of Edinburgh. Data will be analysed thematically with the aid of NVivo qualitative data analysis software.

Economic analysis will be performed by health economists from the University of Edinburgh. The evaluation will assess cost per QALY of PHOENIx relative to standard care in assisting people with SMD.

### Trial steering committee and data monitoring committee

An independently chaired TSC will oversee the PHOENIx RCT. The TSC will meet every other month depending on the needs of the trial. The TSC will have responsibility for advising on practical aspects of the trial, as well as ensuring safety of study participants and providing assurance to the funder and trial sponsor.

A separate Data Monitoring Committee will be set up to monitor data from the trial in order to safeguard the safety and well-being of participants.

### Data management

Paper-based Clinical Report Forms (CRFs) will be completed by site researchers and study data will be collected and managed using Research Electronic Data Capture (REDCap) tools hosted at the University of Edinburgh.^41 42^ REDCap is a secure, web-based software platform designed to support data capture for research studies, providing: (1) An interface for validated data capture, (2) Audit trails for tracking data, (3) Automated export procedures for data downloads and (4) Procedures for data integration and interoperability with external sources. Paper-based CRFs will be transferred to an office in the University of Edinburgh and locked securely in a cabinet within a locked room. Consent forms with identifiable participant data will be kept separate from study CRFs. Data storage and record management requirements, data protection and freedom of information will be compliant with the UK General Data Protection Regulation and the UK Data Protection Act (2018 and subsequent amendments), meaning that the sponsor adheres to policies that are designed to protect the security, accuracy, integrity and confidentiality of personal data.

Qualitative data will be transcribed, pseudonymised and uploaded onto NVivo for the first iteration of the qualitative analysis. All paper-based qualitative data will be subject to the same policies and procedures applicable to quantitative data. Additionally, qualitative interviews will be recorded onto an encrypted data device and stored on the University of Edinburgh electronic server and password protected for added security. Once data is transferred from the data device to the server, data from the data device will be destroyed. Details of interviewees will be pseudonymised to protect anonymity.

## Discussion

The primary care of people who have SMD is provided by different disease-focused health specialities and social care organisations working independently. Reasons for this fragmentation of care are unclear but may be due to the range and severity of the problems experienced by people who have SMD, or their lack of fixed abode. The result is that people who have SMD have difficulty navigating, accessing, co-ordinating and continuing health and social care, adding weight to their already significant treatment burden.^7^ Lacking personal support to obtain primary care, people who have SMD postpone receiving help until it is too late and they present to emergency services, often self-medicating with street drugs leading to overdose.

For over 30 years, published evidence from across the world has described premature mortality and poor health in people experiencing homelessness and other disadvantages.^43^ The causes of death and patterns of morbidity have changed over time, but life expectancy remains worse than most other groups,^36 15^,^17^ suggesting the need for a paradigm shift towards an upstream approach to caring for people who are off the scale of the social hierarchy.^44^ The complex PHOENIx intervention offers a step change in the delivery of primary health and social care for people who have SMD. Through relationship-based care, PHOENIx offers comprehensive, co-ordinated and continuous care on outreach and promises integration rather than fragmentation.

The prevention of street-drug overdose and improvement in other health outcomes in people who have SMD, through a complex intervention aiming to address health and social determinants of health on outreach, has not been tested to date. The PHOENIx intervention has undergone thorough pilot and feasibility testing.^46 12 28^,^33^ The planned definitive, pragmatic RCT with parallel qualitative process and economic evaluation will generate rigorous evidence that may inform a paradigm shift towards integrated health and third sector support on outreach.

The study addresses calls for better evidence of ‘what works’ in inclusion health populations^26 36 40^ informing holistic, clinical generalist approaches to reduce the risk of drug death.

Trauma-informed care is recognised as important in caring for people who experience homelessness and other disadvantages,^25 26^ but the extent to which this approach is included in existing interventions is unclear.^7 22 23^ PHOENIx offers stable, protective relationships (which are a core part of trauma-informed support)^27^ for approximately 9 months.

The study will generate evidence to better understand whether an integrated health and social care intervention without the offer of permanent housing can improve outcomes in people who have SMD.

If outcomes from the trial are favourable, then PHOENIx has the potential to be embedded into routine practice, thus potentially having a transformative impact on people experiencing SMD and services supporting them.

### Study limitations

While the study will be conducted in six geographical areas in Scotland which, like the rest of the UK, is covered by the NHS, findings may not be generalisable to other healthcare systems worldwide.

The rationale for the primary endpoint is based on the continuing challenge of drug deaths, particularly among people living in the most socioeconomically deprived areas. While drug deaths may not pose the largest single cause of deaths among people facing SMD in other countries, the secondary outcomes, in particular hospitalisation, are likely to be of relevance.

Self-report of overdose may be subject to recall bias, although by design, this applies equally to both randomised groups and follow-up overdose incidents will be confirmed by cross-referencing with participants’ health and third sector records. The study timeline of 9 months follow-up is limited by available resources.

### Reporting

A standard protocol items: recommendations for interventional trials reporting checklist is appended in this manuscript as an electronic online supplemental file.

## Ethics and dissemination

Approved by NHS North of Scotland Research Ethics Service: IRAS project ID: 345246. Informed written consent to participate in the study will be taken by experienced independent researchers who have undertaken GCP training. Dissemination of baseline findings is anticipated in the first quarter of 2026, with full results expected in the first quarter of 2027. Findings will be shared with participants, wider third sector homelessness teams, health and care partnerships, then disseminated through peer-reviewed journals and conferences worldwide.

## Supplementary material

10.1136/bmjopen-2025-106640 online supplemental file 1

10.1136/bmjopen-2025-106640 online supplemental file 2

10.1136/bmjopen-2025-106640 online supplemental file 3

10.1136/bmjopen-2025-106640 online supplemental file 4

10.1136/bmjopen-2025-106640 online supplemental file 5

## References

[1] Bramley G, Fitzpatrick S, Sosenko F (2020). Mapping the “hard edges” of disadvantage in England: Adults involved in homelessness, substance misuse, and offending. Geographical J.

[2] Hard Edges Scotland (2019). New conversations about severe & multiple disadvantage. Heriot-Watt University; I-SHERE, Lankelly Chase, The Robertson Trust.

[3] Aldridge RW, Story A, Hwang SW (2018). Morbidity and mortality in homeless individuals, prisoners, sex workers, and individuals with substance use disorders in high-income countries: a systematic review and meta-analysis. Lancet.

[4] Lowrie R, McPherson A, Mair FS (2024). Holistic health and social care outreach for people experiencing homelessness with recent non-fatal overdose in Glasgow, Scotland: the Pharmacist and third sector Homeless charity worker Outreach Engagement Non-medical Independent prescriber Rx (PHOENIx) pilot randomised controlled trial. *bmjph*.

[5] Liu M, Luong L, Lachaud J (2021). Adverse childhood experiences and related outcomes among adults experiencing homelessness: a systematic review and meta-analysis. Lancet Public Health.

[6] Lowrie R, McPherson A, Mair FS (2023). Baseline characteristics of people experiencing homelessness with a recent drug overdose in the PHOENIx pilot randomised controlled trial. Harm Reduct J.

[7] Farmer N, McPherson A, Thomson J (2024). ‘There’s No Hope for Any Kind of Decent Life’: A Qualitative Study to Explore the Perspectives of People Experiencing Homelessness with a Recent Non-Fatal Overdose in Scotland. Br J Soc Work.

[8] Rea J (2023). Social relationships, stigma, and wellbeing through experiences of homelessness in the United Kingdom. J Soc Issue.

[9] Potter LC, Stone T, Swede J (2024). Improving access to general practice for and with people with severe and multiple disadvantage: a qualitative study. Br J Gen Pract.

[10] Zeitler M, Williamson AE, Budd J (2020). Comparing the Impact of Primary Care Practice Design in Two Inner City UK Homelessness Services. J Prim Care Community Health.

[11] Queen AB, Lowrie R, Richardson J (2017). Multimorbidity, disadvantage, and patient engagement within a specialist homeless health service in the UK: an in-depth study of general practice data. BJGP Open.

[12] Lowrie R, Paudyal V, McPherson A (2025). Pharmacy Homeless Outreach Engagement Non-medical Independent Prescribing Rx (PHOENIx) Community Pharmacy-Based Pilot Randomized Controlled Trial. J Urban Health.

[13] Jones C, Mair FS, Williamson AE (2023). Treatment burden for people experiencing homelessness with a recent non-fatal overdose: a questionnaire study. *Br J Gen Pract*.

[14] Coombs T, Abdelkader A, Ginige T (2024). Understanding drug use patterns among the homeless population: A systematic review of quantitative studies. Emerg Trend Drug Addict Health.

[15] National Records of Scotland (2024). Homeless Deaths 2023.

[16] Scottish Government 2023 (2023). Drug related deaths in Scotland in 2023. Drug-related deaths in Scotland in 2023 - National Records of Scotland (NRS).

[17] Scottish Government Suspected drug deaths in Scotland: October to December 2021.

[18] Sordo L, Barrio G, Bravo MJ (2017). Mortality risk during and after opioid substitution treatment: systematic review and meta-analysis of cohort studies. BMJ.

[19] McAuley A, Fraser R, Glancy M (2023). Mortality among individuals prescribed opioid-agonist therapy in Scotland, UK, 2011-20: a national retrospective cohort study. Lancet Public Health.

[20] Strang J, Metrebian N, Lintzeris N (2010). Supervised injectable heroin or injectable methadone versus optimised oral methadone as treatment for chronic heroin addicts in England after persistent failure in orthodox treatment (RIOTT): a randomised trial. Lancet.

[21] Nicholls J, Masterton W, Falzon D (2025). The implementation of safer drug consumption facilities in Scotland: a mixed methods needs assessment and feasibility study for the city of Edinburgh. Harm Reduct J.

[22] Hanlon P, Yeoman L, Gibson L (2018). A systematic review of interventions by healthcare professionals to improve management of non-communicable diseases and communicable diseases requiring long-term care in adults who are homeless. BMJ Open.

[23] Hwang SW, Burns T (2014). Health interventions for people who are homeless. Lancet.

[24] Webb TL, Sniehotta FF, Michie S (2010). Using theories of behaviour change to inform interventions for addictive behaviours. Addiction.

[25] Carver H, Ring N, Miler J (2020). What constitutes effective problematic substance use treatment from the perspective of people who are homeless? A systematic review and meta-ethnography. Harm Reduct J.

[26] (2022). Integrated health and social care for people experiencing homelessness. NICE guideline.

[27] NHS Education for Scotland (2020). National Trauma Transformation Programme.

[28] Johnsen S, Cuthill F, Blenkinsopp J (2021). Outreach-based clinical pharmacist prescribing input into the healthcare of people experiencing homelessness: a qualitative investigation. BMC Health Serv Res.

[29] Lowrie R, Stock K, Lucey S (2021). Pharmacist led homeless outreach engagement and non-medical independent prescribing (Rx) (PHOENIx) intervention for people experiencing homelessness: a non- randomised feasibility study. Int J Equity Health.

[30] Lowrie F, Gibson L, Towle I (2019). A descriptive study of a novel pharmacist led health outreach service for those experiencing homelessness. Int J Pharm Pract.

[31] McPherson A, Paudyal V, Lowrie R (2024). Patient and Public Involvement in Research Evaluating Integrated Care for People Experiencing Homelessness: Findings From the PHOENIx Community Pharmacy Pilot Randomised-Controlled Trial. Health Expect.

[32] Farmer N, McPherson A, Thomson J (2024). Perspectives of people experiencing homelessness with recent non-fatal street drug overdose on the Pharmacist and Homeless Outreach Engagement and Non-medical Independent prescribing Rx (PHOENIx) intervention. PLoS One.

[33] Mackinnon S, Wood K, Cunningham Y (2025). Process evaluation for the Pharmacy Homeless Outreach Engagement Non-medical Independent prescribing Rx (PHOENIx) community pharmacy study. PLoS ONE.

[34] NICE (2023). NICE health technology evaluations: the manual. NICE processes and methods: PMG36.

[35] Johnsen S, Cuthill F, Blenkinsopp J (2019). Qualitative evaluation of clinical pharmacist prescribing input into the care of people experiencing homelessness.

[36] Jagpal P, Saunders K, Plahe G (2020). Research priorities in healthcare of persons experiencing homelessness: outcomes of a national multi-disciplinary stakeholder discussion in the United Kingdom. *Int J Equity Health*.

[37] FEANTSA ETHOS - European Typology on Homelessness and Housing Exclusion.

[38] Hoffmann TC, Glasziou PP, Boutron I (2014). Better reporting of interventions: template for intervention description and replication (TIDieR) checklist and guide. BMJ.

[39] Skivington K, Matthews L, Simpson SA (2021). A new framework for developing and evaluating complex interventions: update of Medical Research Council guidance. BMJ.

[40] Luchenski S, Maguire N, Aldridge RW (2018). What works in inclusion health: overview of effective interventions for marginalised and excluded populations. The Lancet.

[41] Harris PA, Taylor R, Thielke R (2009). Research electronic data capture (REDCap)--a metadata-driven methodology and workflow process for providing translational research informatics support. J Biomed Inform.

[42] Harris PA, Taylor R, Minor BL (2019). The REDCap consortium: Building an international community of software platform partners. J Biomed Inform.

[43] Hibbs JR, Benner L, Klugman L (1994). Mortality in a Cohort of Homeless Adults in Philadelphia. N Engl J Med.

[44] Marmot M (2018). Inclusion health: addressing the causes of the causes. Lancet.

