# Supplementary material for: Protocol for a multicentre randomised controlled trial of the Pharmacy Homeless Outreach Engagement Non-medical and Independent Prescriber (PHOENIx) intervention for people facing severe and multiple disadvantages
Source: BMJ Open. 2025 Nov 23;15(11):e106640. doi: 10.1136/bmjopen-2025-106640 (PMC12645644; doi:10.1136/bmjopen-2025-106640)
Supplement: online supplemental file 1 [file bmjopen-15-11-s001.docx]

**PARTICIPANT CONSENT FORM**

**PHOENIx for people facing Severe and Multiple Disadvantage**

| Name: |  | Date of Birth:  (dd/mmm/yyyy) |  |
| --- | --- | --- | --- |
| Participant ID No: |  | Centre ID: |  |

|  |  | *Please initial box* |
| --- | --- | --- |
| 1. | I confirm that I have read and understood the Participant Information Sheet for the above study. |  |
| 2. | I have been given the opportunity to consider the information provided, ask questions and have had these questions answered to my satisfaction. |  |
| 3. | I understand that my participation is voluntary and that I can ask to withdraw at any time without giving a reason and without my medical care of any support I receive from any other agency being affected. |  |
| 4. | I understand that relevant sections of my medical notes collected during the study may be looked at by individuals from the Sponsors (University of Edinburgh and NHS Lothian), where it is relevant to my taking part in this research. I give permission for these individuals to have access to my data and medical records. |  |
| 5. | I understand that my de-identified data will be stored for at least 10 years and the numerical data may be used in ethically approved research and teaching. |  |
| 6. | I give permission for my personal information (including Community Health Index (CHI) number, initials, name, date of birth, ethnicity, address, postcode, telephone number and consent form) to be retained on NHS and University of Edinburgh servers for administration of this study. |  |
| 7. | I consent for health care, social care, third sector, police and prison, housing providers, rehab services, ambulance service, friends and family to be contacted to disclose my contact information in the event that I cannot be located, e.g. for follow up data collection at 9 months or to check where I live during this period. |  |
|  |  | *Please initial box* |
| 8. | I agree to my General Practitioner (if I am registered) being informed of my participation in the study. |  |
| 9. | If I am in the PHOENIx group, I agree to an NHS Pharmacist or NHS Community Pharmacist looking up my NHS and alcohol/drug recovery service records and making any changes necessary to make sure my medicines and care are the best they can be. |  |
| 10. | If interviewed by a researcher to ask about the PHOENIx intervention, I agree to my interview being audio recorded. |  |
| 11. | If interviewed, I agree to my audio recorded interview being transcribed by a third-party contractor. |  |
| 12. | I agree to the PHOENIx team sharing my identifiable data (not any audio recordings) with services at 9 months, to find out if PHOENIx makes a difference. These services include: NHS (including alcohol/drug services, social care and third sector services, Police Scotland, Scottish Prisons Service, housing services, Councils (homelessness services), rehabilitation providers and National Records Scotland (National Health Service Central Register). |  |
| 13. | I agree to the study team contacting all of the services mentioned in point 12 (above), after 5 and 10 years to find out whether the PHOENIx intervention makes a difference over the long term. |  |
| 14. | I agree to take part in the above study. |  |

| Name of person giving consent | Date | Signature |
| --- | --- | --- |
| __________________________ | ______________ | _______________________________ |
| Name of person taking consent | Date | Signature |
| __________________________ | ______________ | _______________________________ |
