# Supplementary material for: Protocol for a multicentre randomised controlled trial of the Pharmacy Homeless Outreach Engagement Non-medical and Independent Prescriber (PHOENIx) intervention for people facing severe and multiple disadvantages
Source: BMJ Open. 2025 Nov 23;15(11):e106640. doi: 10.1136/bmjopen-2025-106640 (PMC12645644; doi:10.1136/bmjopen-2025-106640)
Supplement: online supplemental file 2 [file bmjopen-15-11-s002.docx]

BASELINE VISIT IN PERSON

Date of Baseline Visit / /

*D D M M M Y Y Y Y*

ELIGIBILITY CRITERIA

| Inclusion Criteria *(all must be answered YES to proceed)* | | | | Yes | | No |
| --- | --- | --- | --- | --- | --- | --- |
| 1. ≥ 18yrs old | | | | ❑ | | ❑ |
| 2. Currently experiencing homelessness (any form of homelessness) * | | | | ❑ | | ❑ |
| 3. At least one street-drug related overdose (self-report or worker/friend) ** | | | | ❑ | | ❑ |
| 4. At least one criminal justice encounter  (arrested/cautioned/charged/imprisoned/DTTO) | | | | ❑ | | ❑ |
|  |  |  |  | |  | |
| Exclusion Criteria *(all must be answered NO to proceed)* | | | | Yes | | No |
| 1. Living in residential accommodation with 24-hour medical care | | | | ❑ | | ❑ |
| 2. Lacking capacity to consent (in the opinion of researcher) | | | | ❑ | | ❑ |
| 3. Posing a safety risk to self/others | | | | ❑ | | ❑ |

***Homeless**: living rough; night shelter; homeless accommodation; women’s shelter; Immigrant accommodation; supported or unsupported temporary accommodation; insecure accommodation; under threat of eviction/violence; temporary non-conventional structures; unfit housing; overcrowding; sofa surfing; ‘No Fixed Abode’

****Overdose:** blackout/ loss of consciousness/Naloxone administered/ attended A&E or hospital for blackout due to drug use/ Ambulance call out due to street drug use.

DEMOGRAPHIC INFORMATION

| Gender: | Male ❑ Female ❑ Trans ❑ Prefer not to say ❑ | | Age: ______ |
| --- | --- | --- | --- |
| Ethnicity: *(please tick one)* | White ❑ Asian/Asian Scottish/Asian British ❑ African Caribbean or Black ❑ Mixed or Multiple Ethnic Groups ❑ Other Ethnic Group ❑ Refused/Not Provided ❑ | | |
| Country of Birth: | _________________________ | | |
| Do you have children? Yes ❑ No ❑ | | How many children under the age of 16 years? ______ | |
| Were you ever in care as a child? Yes ❑ No ❑ | |  | |

CURRENT SERVICE REGISTRATION

| **GP?** | | Yes ❑ | | No ❑ | If yes, practice name and address:  ________________________________________  ________________________________________  ________________________________________ |
| --- | --- | --- | --- | --- | --- |
| Practice Code: ___________________________ | | | | |  |
| How far do you live from your GP?  _________________________________________ | | | | | How do you get there?  _________________________________________ |
| **Alcohol and Drug Recovery Service?** | Yes ❑ | | No ❑ | | If yes, service name and address:  ________________________________________  ________________________________________  ________________________________________ |
| How far do you live from your Alcohol/Drug Recovery Service?  _________________________________________ | | | | | How do you get there?  _________________________________________ |
| **Mental Health Team?** | Yes ❑ | | No ❑ | | If yes, team name and address:  ________________________________________  ________________________________________  ________________________________________ |
| How far do you live from your Mental Health team?  _________________________________________ | | | | | How do you get there?  _________________________________________ |
| **Other teams or services providing support?** | Yes ❑ | | No ❑ | | If yes, describe below:  ________________________________________  ________________________________________ |
| Service name and address:  _________________________________________  _________________________________________  _________________________________________ | | | | | How far do you live from these services?  ________________________________________  How do you get there?  _________________________________________ |

| HEALTH MEASURES | |
| --- | --- |
| Height | cm |
| Weight | kg |
| Blood pressure (sitting) | / mm/Hg |
| Blood pressure (standing) | / mm/Hg |
| Oxygen saturation (O2 sats %) |  |
| Heart Rate (Pulse) |  |
| COPD 6 | FEV1 FEV6 |
|  | Lung age |
| Family History of CHD/Stroke (close relative developed heart disease or had a stroke before reaching 60 yrs?) | Yes No Don’t know |

DIAGNOSES: PHYSICAL HEALTH

| Participant reported: | | | | | |  |
| --- | --- | --- | --- | --- | --- | --- |
| Any allergies (current)? Yes ❑ No ❑ Don’t Know ❑ If yes, details: _________________________ | | | | | | |
| _____________________________________________________________________________________  _____________________________________________________________________________________  _____________________________________________________________________________________  _____________________________________________________________________________________  _____________________________________________________________________________________  _____________________________________________________________________________________ | | | | | |  |
| Gastro-intestinal | ❑ | Cardiovascular | ❑ | Respiratory | ❑ | |
| Nervous | ❑ | Infection | ❑ | Endocrine | ❑ | |
| Genito-urinary | ❑ | Immune System and Malignant Disease | ❑ | Blood & Nutrition | ❑ | |
| Musculoskeletal | ❑ | Eye | ❑ | Ear, Nose & Throat | ❑ | |
| Skin | ❑ |  |  |  |  | |

DIAGNOSES: PHYSICAL HEALTH (continued)

| Any wounds (current)? Yes ❑ No ❑ Don’t Know ❑ | | | |
| --- | --- | --- | --- |
| If yes … | Wound Type(s):  _______________________________  _______________________________  _______________________________  _______________________________  _______________________________  _______________________________ | | Location(s):  _______________________________  _______________________________  _______________________________  _______________________________  _______________________________  _______________________________ |
| Seizures (current)? Yes ❑ No ❑ Don’t Know ❑  If yes, details …  __________________________________________  __________________________________________  __________________________________________ | | Dental Problems (current)? Yes ❑ No ❑ Don’t Know ❑  If yes, details …  _________________________________________  _________________________________________  _________________________________________ | |
| Broken bones/fractures (ever)? Yes ❑ No ❑ Don’t Know ❑  If yes, details …  __________________________________________  __________________________________________  __________________________________________ | | Skin Problems? Yes ❑ No ❑ Don’t Know ❑  If yes, details …  _________________________________________  _________________________________________  _________________________________________ | |
| Head Injuries (ever)? Yes ❑ No ❑ Don’t Know ❑  If yes, details …  __________________________________________  __________________________________________  __________________________________________ | | Assaulted in past year? Yes ❑ No ❑ Don’t Know ❑  If yes, details …  _________________________________________  _________________________________________  _________________________________________ | |
| Currently pregnant? Yes ❑ No ❑ Don’t Know ❑ Not applicable ❑ | | | |

PRESCRIBED MEDICINES

| From case notes: | | | | | |  |
| --- | --- | --- | --- | --- | --- | --- |
| Medicine:  Medicine:  Medicine:  Medicine:  Medicine:  Medicine:  Medicine:  Medicine:  Medicine:  Medicine: | | Dose:  Dose:  Dose:  Dose:  Dose:  Dose:  Dose:  Dose:  Dose:  Dose: | | Frequency:  Frequency:  Frequency:  Frequency:  Frequency:  Frequency:  Frequency:  Frequency:  Frequency:  Frequency: | | |
| Gastro-intestinal | ❑ | Cardiovascular | ❑ | Respiratory | ❑ | |
| Nervous | ❑ | Infection | ❑ | Endocrine | ❑ | |
| Genito-urinary | ❑ | Immune System and Malignant Disease | ❑ | Blood & Nutrition | ❑ | |
| Musculoskeletal | ❑ | Eye | ❑ | Ear, Nose & Throat | ❑ | |
| Skin | ❑ |  |  |  |  | |

BREATHING

| In terms of breathing … (pick only one from below that best describes you) | |  |
| --- | --- | --- |
| Do you get breathless only with hard exercise? | ❑ | |
| Are you breathless when in a rush or walking up a slight hill? | ❑ | |
| Do you walk slower than people who are ages with you because of breathlessness or do you have to stop for breath when walking at your own pace? | ❑ | |
| Do you have to stop for a breath after walking 100 yards on the flat, or after a few minutes? | ❑ | |
| Are you too breathless to leave your accommodation or breathless when dressing? | ❑ | |

MENTAL HEALTH

Any mental health problems (current or past): Yes ❑ No ❑

| Participant reported: | | | | | | |  |
| --- | --- | --- | --- | --- | --- | --- | --- |
| _____________________________________________________________________________________  _____________________________________________________________________________________  _____________________________________________________________________________________  _____________________________________________________________________________________  Tick all that apply (office use only): | | | | | | |  |
| Anxiety | | ❑ | Low mood | ❑ | Psychosis | ❑ | |
| Depression | | ❑ | Bipolar | ❑ | Schizophrenia | ❑ | |
| PTSD/Trauma | | ❑ | Personality Disorder(s) | ❑ | Suicide attempt(s) | ❑ | |
| Other (please note above) | | ❑ |  |  |  |  | |
| Do you feel safe? | Yes ❑ No ❑ Don’t Know ❑ | | | If no, or don’t know, details ….  __________________________________ | | | |

| **Depression Screen** | | | | | | | |  |
| --- | --- | --- | --- | --- | --- | --- | --- | --- |
| Lost interest in things you enjoy?  Yes ❑ No ❑ Don’t Know ❑ | | | | Persistent low mood?  Yes ❑ No ❑ Don’t Know ❑ | | | | |
| Any problems/feelings of (tick all that apply): | | | | | | | | |
| Sleep increase | ❑ | Sleep decrease | ❑ | Activity increase | ❑ | Activity decrease | ❑ | |
| Guilt/worthlessness | ❑ | Appetite changes | ❑ | Fatigue | ❑ | Concentration poor | ❑ | |

| **PHQ4** Over the last two weeks, how often have you been bothered by the following problems? (PLEASE CIRCLE) | | | | |  |
| --- | --- | --- | --- | --- | --- |
|  | Not at all | Several days | More than half the days | Nearly every day | |
| 1. Feeling nervous, anxious or on edge | 0 | 1 | 2 | 3 | |
| 1. Not being able to stop/control worrying | 0 | 1 | 2 | 3 | |
| 1. Little interest or pleasure in doing things | 0 | 1 | 2 | 3 | |
| 1. Feeling down, depressed or hopeless | 0 | 1 | 2 | 3 | |

OVERDOSE (blackout, ambulance, naloxone, hospital)

| Number of overdoses? | | ________ | Roughly, how many of your overdoses needed someone else to help, e.g. ambulance or accommodation staff? | | | ______ |
| --- | --- | --- | --- | --- | --- | --- |
| When was your most recent overdose? | | __________ (dd/mmm/yyyy) | Can you say what made you take the drugs that made you overdose?  What drugs caused your overdose? | | ___________________  ___________________  ___________________ | |
| Is there anything you can think of that might help you reduce your chances of overdosing again? | ___________________  ___________________ | | Got naloxone?  Know how to use it? | Yes ❑ No ❑  Yes ❑ No ❑ | | |

STREET DRUG USE

| Drug Name | Frequency | Last used | Quantity | Route | Since when (approx)  (DD/MMM/YYYY) |
| --- | --- | --- | --- | --- | --- |
| Heroin |  |  |  |  |  |
| Cocaine |  |  |  |  |  |
| Street Valium/Benzos |  |  |  |  |  |
| Gabapentin/ Pregabalin |  |  |  |  |  |
| Cannabis |  |  |  |  |  |
| Spice |  |  |  |  |  |
| Other |  |  |  |  |  |

PRESCRIBED OPIATE SUBSTITUTION / DIAZEPAM

| Currently prescribed opiate substitute? | Yes ❑ No ❑ Don’t Know ❑ |
| --- | --- |
| If yes, please tick all that apply | Methadone ❑ Buprenorphine ❑ Buvidal ❑ Espranor ❑ |
| Dose ____________________________ | Daily ❑ Weekly ❑ Monthly ❑ |
| If not in treatment now, have you ever been in treatment for an opiate problem? | Yes ❑ No ❑ Don’t Know ❑ |
| When (approx) and what treatment? | .….../……./….... _______________________________ |
| Ever had detox/rehab for drug use?  If yes, how many and when? | Yes ❑ No ❑ Don’t Know ❑  How many: ________ / When? ___________ |
| Currently prescribed Diazepam?  If yes, dose and frequency | Yes ❑ No ❑  Dose: ________ / Frequency: _________ |
| If not in treatment now, have you been in treatment for street Diazepam use? | Yes ❑ No ❑ Don’t Know ❑ |

ALCOHOL

| Ever had detox for alcohol?  Yes ❑ No ❑ Don’t know ❑  If yes, on how many occasions? ______________  Ever had rehab for alcohol?  Yes ❑ No ❑ Don’t know ❑  If yes, on how many occasions? ______________ | | Previous hallucinations or seizures with alcohol?  Yes ❑ No ❑ Don’t know ❑ | |
| --- | --- | --- | --- |
| Type of alcohol | __________________ | Amount ____________ | Units ___________ |
| Age of first drink | __________________ | Age first drunk | ________________ |

SMOKING (half ounce = 15g tobacco = 20 cigarettes)

| Current tobacco smoker? | Yes ❑ No ❑ | Ex-smoker? (>10 years ago) | Yes ❑ No ❑ |
| --- | --- | --- | --- |
| Non-smoker? | Yes ❑ No ❑ | If current or ex-smoker, age started | ____________ |
| No of cigarettes per day  No of roll-ups per day | ____________ or  ____________ | Disposable vape?  E-cigarette? | Yes ❑ No ❑  Yes ❑ No ❑ |

DIET – WHAT DO YOU EAT IN A TYPICAL DAY?

| Breakfast | Yes ❑ No ❑ | Lunch | Yes ❑ No ❑ | Dinner | Yes ❑ No ❑ |
| --- | --- | --- | --- | --- | --- |

EXERCISE IN A NORMAL WEEK

| None ❑ | Low (e.g. collect prescription) ❑ | Medium (e.g. walking) ❑ | | High (e.g. gym work) ❑ |
| --- | --- | --- | --- | --- |
| How many times do you exercise in an average week? ____________ | | | Duration __________________ | |

ACCOMMODATION/HOUSING

| Type of accommodation now: ___________________________________________________________  *(tick all that apply)* | | | | | | | |  |
| --- | --- | --- | --- | --- | --- | --- | --- | --- |
| Living rough | ❑ | Homeless accommodation | | ❑ | Immigrant accommodation | | ❑ | |
| Supported Accommodation | ❑ | Night Shelter | | ❑ | Women’s Shelter | | ❑ | |
| Residential Care | ❑ | Unfit housing | | ❑ | Temporary, non-conventional structure | | ❑ | |
| Sofa Surfing | ❑ | Overcrowding | | ❑ | Threat of eviction | | ❑ | |
| Living under threat of violence | ❑ | Other (describe below) | | ❑ |  | | | |
|  |  | _________________________________________________________ | | | | | | |
| Evicted in the last year? | | | Yes ❑ No ❑ Don’t Know ❑ | | | | | |
| Number of times changed accommodation in past year | | | | | | _________________________ | | |
| Number of years homeless | | | | | | _________________________ | | |
| Do you have a support worker? | | | Yes ❑ No ❑ | | | | | |
| If yes, who? | | | Housing ❑ Employment ❑ | | | | | |
| Frequency of visits? | | | _____________________________________________ | | | | | |

ACCOMMODATION/HOUSING (continued)

| Other support? | Yes ❑ No ❑ |
| --- | --- |
| Please state | _____________________________________________  _____________________________________________ |
| What is provided in the housing support you receive? __________________________________________  _______________________________________________________________________________________ | |

EMPLOYMENT/ACTIVITY

| Are you employed now?  Yes ❑ No ❑ / Type of Work ________________ | Have you been employed in the last year?  Yes ❑ No ❑ | |
| --- | --- | --- |
| If employed, type of employment?  Paid ❑ / Voluntary ❑ / Other ❑ | Number of paid jobs  Number of voluntary jobs | _________________  _________________ |
| Do you do any voluntary work now?  Yes ❑ No ❑ | Are you on any training program now?  Yes ❑ No ❑ | |
| Have you been on any training programs in the last year? Yes ❑ No ❑ | Any job/work before being homeless?  Yes ❑ No ❑ / Type of Work ________________ | |
| Do you take part in any structured activity?  Yes ❑ No ❑ | If yes, what type of activity, e.g. sports/gym/art?  _________________________________________ | |
| If you got the chance, what kind of work/activities would you do during the day? | _________________________________________ | |

BENEFITS

| Currently in receipt of benefits? | Yes ❑ No ❑ | Currently applying for/ awaiting decision? | Yes ❑ No ❑ |
| --- | --- | --- | --- |
| Type of current benefits? | ___________________ | Amount/month (approx) | ___________________ |
| Type of current benefits? | ___________________ | Amount/month (approx) | ___________________ |
| Type of current benefits? | ___________________ | Amount/month (approx) | ___________________ |
| Type of current benefits? | ___________________ | Amount/month (approx) | ___________________ |

POLICE HISTORY

| Stopped / Questioned? | Yes ❑ No ❑ | How many times? | 1-10 ❑  51-100 ❑ | 11-50 ❑  100+ ❑ |
| --- | --- | --- | --- | --- |
| Cautioned? | Yes ❑ No ❑ | How many times? | 1-10 ❑  51-100 ❑ | 11-50 ❑  100+ ❑ |
| Charged? | Yes ❑ No ❑ | How many times? | 1-10 ❑  51-100 ❑ | 11-50 ❑  100+ ❑ |
| Convicted of a crime? | Yes ❑ No ❑ | How many times? | 1-10 ❑  51-100 ❑ | 11-50 ❑  100+ ❑ |
| Custodial sentence? | Yes ❑ No ❑ | How long? | ___________________ | |
| Suspended sentence? | Yes ❑ No ❑ | How long? | ___________________ | |
| Drug Treatment & Testing Order (DTTO)? | Yes ❑ No ❑ | How long? | ___________________ | |

QUALITY OF LIFE (EQ-5D-5L)

| Under each heading, please tick the ONE box that best describes your health TODAY. | |
| --- | --- |
| **MOBILITY** |  |
| I have no problems in walking about | ❑ |
| I have slight problems in walking about | ❑ |
| I have moderate problems in walking about | ❑ |
| I have severe problems in walking about | ❑ |
| I am unable to walk about | ❑ |
| **SELF-CARE** |  |
| I have no problems washing or dressing myself | ❑ |
| I have slight problems washing or dressing myself | ❑ |
| I have moderate problems washing or dressing myself | ❑ |
| I have severe problems washing or dressing myself | ❑ |
| I am unable to wash or dress myself | ❑ |
| **USUAL ACTIVITIES** *(e.g. work, study, housework, family or leisure activities)* |  |
| I have no problems doing my usual activities | ❑ |
| I have slight problems doing my usual activities | ❑ |
| I have moderate problems doing my usual activities | ❑ |
| I have severe problems doing my usual activities | ❑ |
| I am unable to do my usual activities | ❑ |
| **PAIN / DISCOMFORT** |  |
| I have no pain or discomfort | ❑ |
| I have slight pain or discomfort | ❑ |
| I have moderate pain or discomfort | ❑ |
| I have severe pain or discomfort | ❑ |
| I have extreme pain or discomfort | ❑ |
| **ANXIETY / DEPRESSION** |  |
| I am not anxious or depressed | ❑ |
| I am slightly anxious or depressed | ❑ |
| I am moderately anxious or depressed | ❑ |
| I am severely anxious or depressed | ❑ |
| I am extremely anxious or depressed | ❑ |

10

0

20

30

40

50

60

80

70

90

100

5

15

25

35

45

55

75

65

85

95

We want to know how good or bad your health is today.

On a scale of 0 (worst) to 100 (best possible health), your Health Number Today is: ______

How do you think you could improve this number? _________________________________

RANDOMISATION

|  |  |  |
| --- | --- | --- |
| Phone call to randomisation line | Yes ❑ | No ❑ |

| Please indicate the participant’s allocation | | |
| --- | --- | --- |
| 1 – PHOENIx Intervention plus usual care  2 – Usual Care alone |  | ❑  ❑ |

|  |  |  |
| --- | --- | --- |
| *Randomisation completed by Signature* | *Print Name* | *Date (DD/MMM/YYYY)* |

VISIT REVIEW

|  | | |  | |  |
| --- | --- | --- | --- | --- | --- |
| Visit completed as per protocol  *If no record protocol non-compliance as required* | | Yes ❑ | | No ❑ | |
| Visit and allocation sent to GP | | Yes ❑ | | No ❑ | |
| Next visit booked for | / / *(DD/MMM/YYYY)* | | | | |
| Comments: |  | | | | |

|  |  |  |
| --- | --- | --- |
| *Completed by Signature* | *Print Name* | *Date (DD/MM/YYYY)* |

| Voucher given? | Yes ❑ | No ❑ |
| --- | --- | --- |
