# Supplementary material for: Protocol for a multicentre randomised controlled trial of the Pharmacy Homeless Outreach Engagement Non-medical and Independent Prescriber (PHOENIx) intervention for people facing severe and multiple disadvantages
Source: BMJ Open. 2025 Nov 23;15(11):e106640. doi: 10.1136/bmjopen-2025-106640 (PMC12645644; doi:10.1136/bmjopen-2025-106640)
Supplement: online supplemental file 3 [file bmjopen-15-11-s003.docx]

FOLLOW-UP VISIT AT 9 MONTHS IN PERSON

Date of Baseline Interview / /

*D D M M M Y Y Y Y*

Date of Follow-Up Visit / /

*D D M M M Y Y Y Y*

| Visit completed: | Face-to- Face | ❑ | Remote via teams | ❑ | Remote via tel | ❑ |
| --- | --- | --- | --- | --- | --- | --- |

CURRENT SERVICE REGISTRATION

| **GP?** | | Yes ❑ | | No ❑ | If yes, practice name and address:  ________________________________________  ________________________________________  ________________________________________ |
| --- | --- | --- | --- | --- | --- |
| Practice Code: ___________________________ | | | | |  |
| How far do you live from your GP?  _________________________________________ | | | | | How do you get there?  _________________________________________ |
| **Alcohol and Drug Recovery Service?** | Yes ❑ | | No ❑ | | If yes, service name and address:  ________________________________________  ________________________________________  ________________________________________ |
| How far do you live from your Alcohol/Drug Recovery Service?  _________________________________________ | | | | | How do you get there?  _________________________________________ |
| **Mental Health Team?** | Yes ❑ | | No ❑ | | If yes, team name and address:  ________________________________________  ________________________________________  ________________________________________ |
| How far do you live from your Mental Health team?  _________________________________________ | | | | | How do you get there?  _________________________________________ |

CURRENT SERVICE REGISTRATION (continued)

| **Other teams or services providing support?** | Yes ❑ | No ❑ | If yes, describe below:  ________________________________________  ________________________________________ |
| --- | --- | --- | --- |
| Service name and address:  _________________________________________  _________________________________________  _________________________________________ | | | How far do you live from these services?  ________________________________________  How do you get there?  _________________________________________ |

| HEALTH MEASURES | |
| --- | --- |
| Weight | kg |
| Blood pressure (sitting) | / mm/Hg |
| Blood pressure (standing) | / mm/Hg |
| Oxygen saturation (O2 sats %) |  |
| Heart Rate (Pulse) |  |
| COPD 6 | FEV1 FEV6 |
|  | Lung age |

DIAGNOSES: PHYSICAL HEALTH

| Participant reported: | | | | | | | | |  |
| --- | --- | --- | --- | --- | --- | --- | --- | --- | --- |
| Any allergies (since baseline)? Yes ❑ No ❑ Don’t Know ❑ If yes, details: ____________________ | | | | | | | | | |
| _____________________________________________________________________________________  _____________________________________________________________________________________  _____________________________________________________________________________________  _____________________________________________________________________________________ | | | | | | | | |  |
| Gastro-intestinal | | ❑ | Cardiovascular | | ❑ | | Respiratory | ❑ | |
| Nervous | | ❑ | Infection | | ❑ | | Endocrine | ❑ | |
| Genito-urinary | | ❑ | Immune System and Malignant Disease | | ❑ | | Blood & Nutrition | ❑ | |
| Musculoskeletal | | ❑ | Eye | | ❑ | | Ear, Nose & Throat | ❑ | |
| Skin | | ❑ |  | |  | |  |  | |
| Any wounds (current)? Yes ❑ No ❑ Don’t Know ❑ | | | | | | | | | |
| If yes … | Wound Type(s):  _______________________________  _______________________________  _______________________________ | | | | | Location(s):  _______________________________  _______________________________  _______________________________ | | | |
| Seizures (current)? Yes ❑ No ❑ Don’t Know ❑ / If yes, details …  __________________________________________  __________________________________________ | | | | Dental Problems (current)? Yes ❑ No ❑ Don’t Know ❑ / If yes, details …  _________________________________________  _________________________________________ | | | | | |
| Broken bones/fractures (since baseline visit)? Yes ❑ No ❑ Don’t Know ❑ / If yes, details …  __________________________________________  __________________________________________ | | | | Skin Problems? Yes ❑ No ❑ Don’t Know ❑ / If yes, details …  _________________________________________  _________________________________________ | | | | | |
| Head Injuries (since baseline visit)? Yes ❑ No ❑ Don’t Know ❑ / If yes, details …  __________________________________________  __________________________________________ | | | | Assaulted since baseline visit? Yes ❑ No ❑ Don’t Know ❑ / If yes details …  _________________________________________  _________________________________________ | | | | | |
| Currently pregnant? Yes ❑ No ❑ Don’t Know ❑ Not applicable ❑ | | | | | | | | | |

PRESCRIBED MEDICINES

| From case notes: | | | | | |  |
| --- | --- | --- | --- | --- | --- | --- |
| Medicine:  Medicine:  Medicine:  Medicine:  Medicine:  Medicine:  Medicine:  Medicine:  Medicine:  Medicine: | | Dose:  Dose:  Dose:  Dose:  Dose:  Dose:  Dose:  Dose:  Dose:  Dose: | | Frequency:  Frequency:  Frequency:  Frequency:  Frequency:  Frequency:  Frequency:  Frequency:  Frequency:  Frequency: | | |
| Gastro-intestinal | ❑ | Cardiovascular | ❑ | Respiratory | ❑ | |
| Nervous | ❑ | Infection | ❑ | Endocrine | ❑ | |
| Genito-urinary | ❑ | Immune System and Malignant Disease | ❑ | Blood & Nutrition | ❑ | |
| Musculoskeletal | ❑ | Eye | ❑ | Ear, Nose & Throat | ❑ | |
| Skin | ❑ |  |  |  |  | |

BREATHING

| In terms of breathing … (pick only one from below that best describes you) | |  |
| --- | --- | --- |
| Do you get breathless only with hard exercise? | ❑ | |
| Are you breathless when in a rush or walking up a slight hill? | ❑ | |
| Do you walk slower than people who are ages with you because of breathlessness or do you have to stop for breath when walking at your own pace? | ❑ | |
| Do you have to stop for a breath after walking 100 yards on the flat, or after a few minutes? | ❑ | |
| Are you too breathless to leave your accommodation or breathless when dressing? | ❑ | |

MENTAL HEALTH

Any mental health problems (current): Yes ❑ No ❑

| Participant reported: | | | | | | |  |
| --- | --- | --- | --- | --- | --- | --- | --- |
| _____________________________________________________________________________________  _____________________________________________________________________________________  _____________________________________________________________________________________  _____________________________________________________________________________________  Tick all that apply (office use only): | | | | | | |  |
| Anxiety | | ❑ | Low mood | ❑ | Psychosis | ❑ | |
| Depression | | ❑ | Bipolar | ❑ | Schizophrenia | ❑ | |
| PTSD/Trauma | | ❑ | Personality Disorder(s) | ❑ | Suicide attempt(s) | ❑ | |
| Other (please note above) | | ❑ |  |  |  |  | |
| Do you feel safe? | Yes ❑ No ❑ Don’t Know ❑ | | | If no, or don’t know, details ….  __________________________________ | | | |

| **Depression Screen** | | | | | | | |  |
| --- | --- | --- | --- | --- | --- | --- | --- | --- |
| Lost interest in things you enjoy?  Yes ❑ No ❑ Don’t Know ❑ | | | | Persistent low mood?  Yes ❑ No ❑ Don’t Know ❑ | | | | |
| Any problems/feelings of (tick all that apply): | | | | | | | | |
| Sleep increase | ❑ | Sleep decrease | ❑ | Activity increase | ❑ | Activity decrease | ❑ | |
| Guilt/worthlessness | ❑ | Appetite changes | ❑ | Fatigue | ❑ | Concentration poor | ❑ | |

| **PHQ4** Over the last two weeks, how often have you been bothered by the following problems? (PLEASE CIRCLE) | | | | |  |
| --- | --- | --- | --- | --- | --- |
|  | Not at all | Several days | More than half the days | Nearly every day | |
| 1. Feeling nervous, anxious or on edge | 0 | 1 | 2 | 3 | |
| 1. Not being able to stop/control worrying | 0 | 1 | 2 | 3 | |
| 1. Little interest or pleasure in doing things | 0 | 1 | 2 | 3 | |
| 1. Feeling down, depressed or hopeless | 0 | 1 | 2 | 3 | |

OVERDOSE (blackout, ambulance, naloxone, hospital)

| Have you overdosed since our first visit (9 months ago)?  If yes, can you remember the approximate date of the first overdose after our first visit about 9 months ago? | | Yes ❑ No ❑  ___________ (dd/mmm/yyyy) | Number of overdoses since Baseline?  Roughly, how many of your overdoses since Baseline needed someone else to help, e.g. ambulance or accommodation staff? | | | ______  ______ |
| --- | --- | --- | --- | --- | --- | --- |
| When was your most recent overdose? | | __________ (dd/mmm/yyy) | Can you say what made you take the drugs that made you overdose?  What drugs caused your overdose? | | ___________________  ___________________  ___________________ | |
| Is there anything you can think of that might help you reduce your chances of overdosing again? | ___________________  ___________________ | | Got naloxone?  Know how to use it? | Yes ❑ No ❑  Yes ❑ No ❑ | | |

STREET DRUG USE

| Drug Name | Frequency | Last used | Quantity | Route | Since when (approx)  (DD/MMM/YYYY) |
| --- | --- | --- | --- | --- | --- |
| Heroin |  |  |  |  |  |
| Cocaine |  |  |  |  |  |
| Street Valium/Benzos |  |  |  |  |  |
| Gabapentin/ Pregabalin |  |  |  |  |  |
| Cannabis |  |  |  |  |  |
| Spice |  |  |  |  |  |
| Other |  |  |  |  |  |

PRESCRIBED OPIATE SUBSTITUTION / DIAZEPAM

| Currently prescribed opiate substitute? | Yes ❑ No ❑ Don’t Know ❑ |
| --- | --- |
| If yes, please tick all that apply | Methadone ❑ Buprenorphine ❑ Buvidal ❑ Espranor ❑ |
| Dose ____________________________ | Daily ❑ Weekly ❑ Monthly ❑ |
| If not in treatment now, have you been in treatment for an opiate problem in the past 9 months? | Yes ❑ No ❑ Don’t Know ❑ |
| When (approx) and what treatment? | .….../……./….... _______________________________ |
| Had detox/rehab for drug use in the last nine months or since baseline?  If yes, how many and when? | Yes ❑ No ❑ Don’t Know ❑  How many: ________ / When? ___________ |
| Currently prescribed Diazepam?  If yes, dose and frequency | Yes ❑ No ❑  Dose: ____________ / Frequency: _________ |
| If not in treatment now, have you been in treatment for street Diazepam use in the past 9 months? | Yes ❑ No ❑ Don’t Know ❑ |

ALCOHOL

| Any detox for alcohol in past 9 months?  Yes ❑ No ❑ Don’t know ❑  If yes, how many occasions? __________________  Any rehab for alcohol in past 9 months?  Yes ❑ No ❑ Don’t know ❑  If yes, how many occasions? __________________ | Any hallucinations or seizures on alcohol withdrawal in past 9 months?  Yes ❑ No ❑ Don’t know ❑ | |
| --- | --- | --- |
|  | Type of alcohol  How much per week?  Units? | ___________________  ___________________  ___________________ |

SMOKING (half ounce = 15g tobacco = 20 cigarettes)

| Current tobacco smoker? | Yes ❑ No ❑ | Non-smoker? | Yes ❑ No ❑ |
| --- | --- | --- | --- |
| No of cigarettes per day  No of roll-ups per day | ____________ or  ____________ | Disposable vape?  E-cigarette? | Yes ❑ No ❑  Yes ❑ No ❑ |

DIET – WHAT DO YOU EAT IN A TYPICAL DAY?

| Breakfast | Yes ❑ No ❑ | Lunch | Yes ❑ No ❑ | Dinner | Yes ❑ No ❑ |
| --- | --- | --- | --- | --- | --- |

EXERCISE IN A NORMAL WEEK

| None ❑ | Low (e.g. collect prescription) ❑ | Medium (e.g. walking) ❑ | | High (e.g. gym work) ❑ |
| --- | --- | --- | --- | --- |
| How many times do you exercise in an average week? ____________ | | | Duration __________________ | |

ACCOMMODATION/HOUSING

| Type of accommodation now: ___________________________________________________________  *(tick all that apply)* | | | | | | |  |
| --- | --- | --- | --- | --- | --- | --- | --- |
| Living rough | ❑ | Homeless accommodation | | ❑ | Immigrant accommodation | ❑ | |
| Supported Accommodation | ❑ | Night Shelter | | ❑ | Women’s Shelter | ❑ | |
| Residential Care | ❑ | Unfit housing | | ❑ | Temporary, non-conventional structure | ❑ | |
| Sofa Surfing | ❑ | Overcrowding | | ❑ | Threat of eviction | ❑ | |
| Living under threat of violence | ❑ | Other (describe below) | | ❑ |  | | |
|  |  | _________________________________________________________ | | | | | |
| Evicted since Baseline?  If yes, how many times? | | | Yes ❑ No ❑ Don’t Know ❑  ___________________________________________ | | | | |
| Have you changed accommodation since Baseline? | | | Yes ❑ No ❑ Don’t Know ❑ | | | | |
| Number of times changed accommodation since Baseline? | | | | | ________________________ | | |
| What kind of support did you have at each place? | | | | | | | |
| 1. _________________________  _________________________ | | 2. _________________________  _________________________ | | | 3. _________________________  _________________________ | | |
| Do you have a support worker? | | | Yes ❑ No ❑ | | | | |
| If yes, who? Housing ❑ Employment ❑ Other ❑ (please state) _____________________________ | | | | | | | |
| Frequency of visits?  What is provided in the support you receive? | | | ___________________________________________  ___________________________________________ | | | | |

EMPLOYMENT/ACTIVITY

| Are you employed now?  Yes ❑ No ❑ / Type of Work ________________ | Have you been employed since Baseline?  Yes ❑ No ❑ | |
| --- | --- | --- |
| If employed, type of employment?  Paid ❑ / Voluntary ❑ / Other ❑ | Number of paid jobs  Number of voluntary jobs | _________________  _________________ |
| Do you do any voluntary work now?  Yes ❑ No ❑ | Are you on any training program now?  Yes ❑ No ❑ | |
| Have you been on any training programme(s) since Baseline? Yes ❑ No ❑ |  | |
| Do you take part in any structured activity?  Yes ❑ No ❑ | If yes, what type of activity, e.g. sports/gym/art?  _________________________________________  _________________________________________ | |
| If you got the chance, what kind of work/activities would you do during the day? | _________________________________________  _________________________________________ | |

BENEFITS

| Currently in receipt of benefits? | Yes ❑ No ❑ | Currently applying for/ awaiting decision? | Yes ❑ No ❑ |
| --- | --- | --- | --- |
| Type of current benefits? | ___________________ | Amount/month (approx) | ___________________ |
| Type of current benefits? | ___________________ | Amount/month (approx) | ___________________ |
| Type of current benefits? | ___________________ | Amount/month (approx) | ___________________ |
| Type of current benefits? | ___________________ | Amount/month (approx) | ___________________ |

POLICE HISTORY

| Stopped / Questioned? | Yes ❑ No ❑ | How many times? | 1-10 ❑  51-100 ❑ | 11-50 ❑  100+ ❑ |
| --- | --- | --- | --- | --- |
| Cautioned? | Yes ❑ No ❑ | How many times? | 1-10 ❑  51-100 ❑ | 11-50 ❑  100+ ❑ |
| Charged? | Yes ❑ No ❑ | How many times? | 1-10 ❑  51-100 ❑ | 11-50 ❑  100+ ❑ |
| Convicted of a crime? | Yes ❑ No ❑ | How many times? | 1-10 ❑  51-100 ❑ | 11-50 ❑  100+ ❑ |
| Custodial sentence? | Yes ❑ No ❑ | How long? | ___________________ | |
| Suspended sentence? | Yes ❑ No ❑ | How long? | ___________________ | |
| Drug Treatment & Testing Order (DTTO)? | Yes ❑ No ❑ | How long? | ___________________ | |

QUALITY OF LIFE (EQ-5D-5L)

| Under each heading, please tick the ONE box that best describes your health TODAY. | |
| --- | --- |
| **MOBILITY** |  |
| I have no problems in walking about | ❑ |
| I have slight problems in walking about | ❑ |
| I have moderate problems in walking about | ❑ |
| I have severe problems in walking about | ❑ |
| I am unable to walk about | ❑ |
| **SELF-CARE** |  |
| I have no problems washing or dressing myself | ❑ |
| I have slight problems washing or dressing myself | ❑ |
| I have moderate problems washing or dressing myself | ❑ |
| I have severe problems washing or dressing myself | ❑ |
| I am unable to wash or dress myself | ❑ |
| **USUAL ACTIVITIES** *(e.g. work, study, housework, family or leisure activities)* |  |
| I have no problems doing my usual activities | ❑ |
| I have slight problems doing my usual activities | ❑ |
| I have moderate problems doing my usual activities | ❑ |
| I have severe problems doing my usual activities | ❑ |
| I am unable to do my usual activities | ❑ |
| **PAIN / DISCOMFORT** |  |
| I have no pain or discomfort | ❑ |
| I have slight pain or discomfort | ❑ |
| I have moderate pain or discomfort | ❑ |
| I have severe pain or discomfort | ❑ |
| I have extreme pain or discomfort | ❑ |
| **ANXIETY / DEPRESSION** |  |
| I am not anxious or depressed | ❑ |
| I am slightly anxious or depressed | ❑ |
| I am moderately anxious or depressed | ❑ |
| I am severely anxious or depressed | ❑ |
| I am extremely anxious or depressed | ❑ |

10

0

20

30

40

50

60

80

70

90

100

5

15

25

35

45

55

75

65

85

95

We want to know how good or bad your health is today.

On a scale of 0 (worst) to 100 (best possible health), your Health Number Today is: ______

How do you think you could improve this number? _________________________________

VISIT REVIEW

|  | | |  | |  |
| --- | --- | --- | --- | --- | --- |
| Visit completed as per protocol  *If no record protocol non-compliance as required* | | Yes ❑ | | No ❑ | |
| Comments: |  | | | | |

|  |  |  |
| --- | --- | --- |
| *Completed by Signature* | *Print Name* | *Date (DD/MM/YYYY)* |

| Voucher given? | Yes ❑ | No ❑ |
| --- | --- | --- |
