# Supplementary material for: Protocol for a multicentre randomised controlled trial of the Pharmacy Homeless Outreach Engagement Non-medical and Independent Prescriber (PHOENIx) intervention for people facing severe and multiple disadvantages
Source: BMJ Open. 2025 Nov 23;15(11):e106640. doi: 10.1136/bmjopen-2025-106640 (PMC12645644; doi:10.1136/bmjopen-2025-106640)
Supplement: Supplementary file 6 [file bmjopen-2025-106640.reviewer_comments.pdf]

PEER REVIEW HISTORY

BMJ Open publishes all reviews undertaken for accepted manuscripts. Reviewers are asked to complete a checklist review form and are provided with free text boxes to elaborate on their assessment. These free text comments are reproduced below.

ARTICLE DETAILS

Title (Provisional)

Protocol for a multicentre randomised controlled trial of the Pharmacy Homeless Outreach Engagement Non-medical and Independent prescriber (PHOENIx) intervention for people facing severe and multiple disadvantages.

Authors

Lowrie, Richard; McPherson, Andrew; Moir, Jane; McGilvery, Emma; Vickery, Katherine; O'Loan, Jen; Rushworth, Gordon; Paudyal, Vibhu; Adam, Alex; Thomson, Elaine; Rowe, Alison; Ali Akbar, Hannah; Murphy, John; Budd, John; Raeburn, Fiona; Marshall, Trudi; Nelson, Kirsty; Garstka, Zofia; McKinney, Emma; Melville, Lauren; Duncan, Graeme; Breingan, Zoe; Johnsen, Sarah; Stoddart, Andrew; Lewis, Steff; Williamson, Andrea E; Lilley, Jack; Orr, Tracy; Orr, Michael; Kelly, Shona; Macaulay, Mairi; Maclean, Alison; Kennedy, Heather; Sutherland, Andrea; MacLean, Gillian; Rosinska, Magda; Dos Santos, Carlos; Esson, Kelly; Robertson, Craig; Carnegie, Jill; Cortez, Mariangela Alejandro; Orr, Marion

---

This article was not externally reviewed at BMJ Open. Protocols that have been independently assessed prior to submission to BMJ Open are usually fast-tracked to publication on the grounds that further substantial changes will not be possible. This independent assessment will usually be external, independent review for both a substantial grant award from a non-commercial or government funder and ethics approval.
